# Photonic instantaneous frequency measurement of wideband microwave signals

**DOI:** 10.1371/journal.pone.0182231

**Published:** 2017-08-03

**Authors:** Yueqin Li, Li Pei, Jing Li, Yiqun Wang, Jin Yuan, Tigang Ning

**Affiliations:** 1 Key Laboratory of All Optical Network & Advanced Telecommunication Network of Ministry of Education, Institute of Lightwave Technology, Beijing Jiaotong University, Beijing, China; 2 Department of Electrical Engineering, University of California, Los Angeles, California, United States of America; 3 California NanoSystems Institute, Los Angeles, California, United States of America; Ludwig-Maximilians-Universitat Munchen, GERMANY

## Abstract

We propose a photonic system for instantaneous frequency measurement (IFM) of wideband microwave signals with a tunable measurement range and resolution based on a polarization-maintaining fiber Bragg grating (PM-FBG). Firstly, in order to be insensitive to laser power fluctuation, we aim at generating two different frequency to amplitude characteristics so that we can normalize them to obtain an amplitude comparison function (ACF). Then we encode these two different wavelengths in two perpendicular polarizations by using the PM-FBG which shows different transmission profiles at two polarizations. The ACF is capable of being adjusted by tuning polarization angle, therefore the measurement range and resolution are tunable. By theoretical analyses and simulated verification, a frequency measurement range of 0~17.2 GHz with average resolution of ±0.12 GHz can be achieved, which signifies a wide measurement range with relatively high resolution. Our system does not require large optical bandwidth for the components because the wavelength spacing can be small, making the system affordable, stable, and reliable with more consistent characteristics due to the narrowband nature of the optical parts. PM-FBG with high integration can be potentially used for more polarization manipulating systems and the use of a single-polarization dual-wavelength laser can simplify the architecture and enhance the stability.

## Introduction

Instantaneous frequency measurement (IFM) of microwave signals is extensively used in the field of electronic warfare and wireless commutations. With the growing requirements of large bandwidth and applications in the complex electro-magnetic environment, conventional electrical IFM technology is practically limited due to the electronic bottleneck. Thus, photonic technology is proposed and applied to achieve wideband IFM by virtue of its distinct advantages, such as high bandwidth, low power loss and immunity to electro-magnetic interference. In recent years, many approaches have been proposed to implement photonic wideband IFM. According to the mapping modes, the IFM system can be achieved by frequency-to-space mapping [1, 2], frequency-to-time mapping [3] and frequency-to-amplitude mapping. Among them, the approaches [4–18] based on frequency-to-amplitude mapping are most widely studied by researchers. In these systems, by utilizing an amplitude comparison function (ACF) which is derived by comparing two frequency to amplitude characteristics, microwave frequency can be calculated. In the approaches such as [4–8], the IFM system is realized by using different modulators or different dispersive media. The ACF curve can be also obtained by detecting the optical power output from a fiber Bragg grating [9], which possesses high measurement resolution.

However, as always, there is a trade-off between measurement range and resolution. The wider the bandwidth of the instantaneous radio frequency estimation, the less accurate the measurement becomes. To overcome this problem, several photonic IFM techniques with tunable measurement range and resolution have been proposed. One popular solution is by carefully tuning the wavelength of the laser [10, 11], so that the ACF can be adjusted. An approach employing two dispersive media to simultaneously generate multiple ACFs can also extend the measurement range and improve the resolution [12]. The measurement range is adjustable due to the dispersion variation.

When large measurement range is demanded, the wavelength spacing of lasers has to be far apart, so as to get a decent ACF. Researchers have started to explore other tuning mechanisms, which substitute for shifting the laser wavelength. A reconfigurable IFM system based on stimulated Brillouin scattering has been reported in [13]. The measurement range and the resolution are tunable by varying the reference driving frequency. Another reconfigurable IFM system based on a dual-parallel Mach Zehnder modulator (DP-MZM) and a Mach-Zehnder modulator [14] has also been proposed while the measurement range is tuned by adjusting the DC bias voltage. With bias voltage control, tunable IFM system can be realized by using one DP-MZM [15] or a polarization modulator (PolM) [16] as well, achieving high resolution for frequency measurement. Moreover, tuning polarization angle is also a good choice for tunable IFM system with high flexibility. In [17], by simply adjusting a polarization controller after a PolM, the measurement range and resolution can be tuned finely. But the light waves from two lasers are with the same polarization directions and the power fading functions only have slight difference. This calls for a large wavelength spacing regarding to the lasers. Then we have proposed a simplified IFM prototype based on a single laser source and filter-less architecture [18]. However, the walk off effect in the dispersion compensating fiber is enhanced due to the impact of the two different polarization states.

In this paper, we propose a photonic IFM system with tunable measurement range and resolution in order to address the above problems. To obtain significantly different frequency to amplitude characteristics at two laser wavelengths and generate a sensitive ACF, we encode these wavelengths in two perpendicular polarizations. A polarization-maintaining fiber Bragg grating (PM-FBG), which has distinctive transmission profiles at two polarizations, manipulates these two wavelength components independently, despite their small wavelength spacing. Firstly, two wavelength components are modulated by a PolM. Then one of their optical carriers is filtered out along the two orthogonally-polarized transmission bands of PM-FBG separately. By adjusting the polarization angle before the PolM, the ACF curve can be shifted so that the measurement range is tunable. Our approach eliminates the requirement of shifting laser wavelengths, commonly used in other tunable IFM systems. The measurement resolution is also improved by dividing the whole measurement range into several sections. Since the wavelength spacing in a single-polarization dual-wavelength laser is small, there is no requirement for large optical bandwidth for the components. Therefore, the system is affordable, stable, and reliable with consistent characteristics due to its narrowband nature. Owing to the birefringence effect and high integration, PM-FBG exhibits promising applications in many polarization manipulating systems.

## Methods

**Fig 1:**
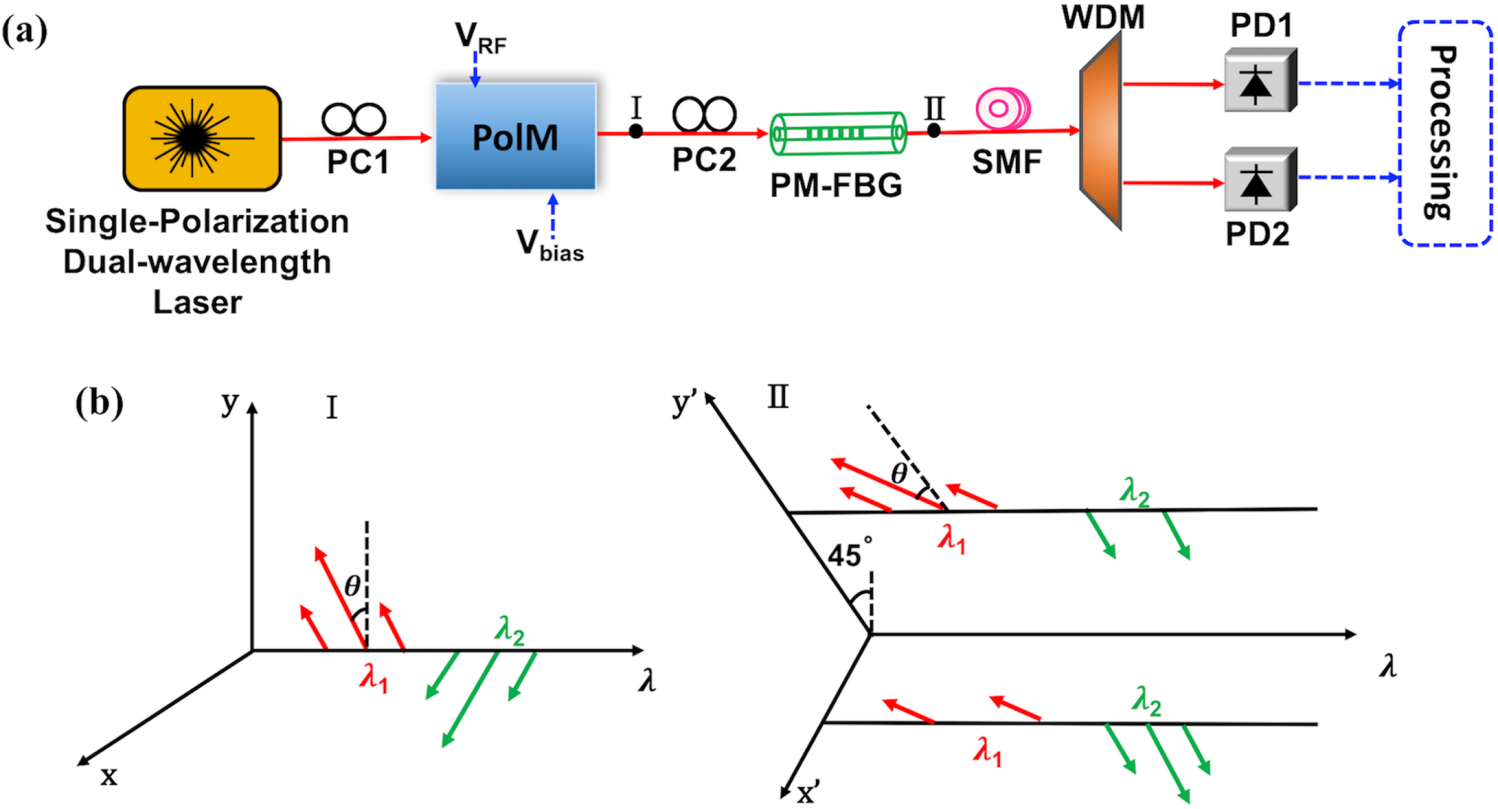
Schematic of the proposed IFM system. (a) The system consists of a single-polarization dual-wavelength laser, two polarization controllers (PCs), a polarization modulator (PolM), a polarization-maintaining Bragg grating (PM-FBG), a single mode fiber (SMF), a wavelength division multiplexer (WDM), two photodetectors (PDs) and a processing unit. The modulator is driven by a voltage of radio frequency (*V*_*RF*_) and is adjusted by a DC bias voltage (*V*_*bias*_). (b) Evolution happens from location I to II in optical spectra and polarization (x and y axes are the principal axes of PolM, xʹ and y′ axes are the fast and slow axes of PM-FBG).

The schematic setup of the proposed IFM system with tunable measurement range and resolution is shown in (Fig 1A). It consists of a single-polarization dual-wavelength laser, two polarization controllers (PCs), a PolM, a PM-FBG, a section of single mode fiber (SMF), a wavelength division multiplexer (WDM), two photodetectors (PDs) and a processing unit. We mark two output locations after modulator and PM-FBG as I and II, then the evolution of their optical spectra and polarization is illustrated in (Fig 1B).

To obtain two wavelength components from the laser, conventionally two lasers are required. But this IFM system employs only one laser with single-polarization and dual-wavelength [19–21]. The two wavelength components output from the laser are orthogonally polarized with a wavelength spacing. One significant advantage of using this laser lies in its capability of being operated in a dual-wavelength mode of single polarization per wavelength, which has a very good stability. The amplitude variation can be smaller than 1.5dB, even as better as 0.5dB [19]. The wavelength variation can be less than 0.01nm [21]. Even if a change in the temperature of the laser will shift the two wavelengths simultaneously, the wavelength spacing and stability will not be affected [19, 20]. Additionally, environmental effects and aging processes act likewise on both wavelengths of this dual-wavelength laser. Thus, the laser is potentially stable while portable, and wavelength tunable while cost effective.

Then PC1 is used to align these two wavelengths with a polarization angle *θ* before they are coupled to the PolM. The modulator is driven by the unknown microwave signal, so that two wavelength components can be modulated with complementary phase modulation. We assume the two principal axes of PolM are x and y, which is shown at location I of (Fig 1B). The output field can be written as

$$\begin{array}{c} \left[\begin{array}{c} E_{x}\\ E_{y} \end{array}\right]\propto \hat{x}(E_{1}e^{j\omega_{1}t}\sin\theta +E_{2}e^{j\omega_{2}t}\cos\theta )\sum_{n=-1}^{1}J_{n}(\beta )e^{j(n\omega t+j\varphi_{0})}\\ +\hat{y}(E_{1}e^{j\omega_{1}t}\cos\theta -E_{2}e^{j\omega_{2}t}\sin\theta )\sum_{n=-1}^{1}J_{n}(-\beta )e^{jn\omega t} \end{array}$$ (1)

where *E*_*1*_ and *E*_*2*_ are magnitude of optical carriers, *ω*_*1*_ and *ω*_*2*_ are angular frequency of two optical carriers, *J*_*n*_ is Bessel function of the first kind of order n, *φ*_*0*_ = *πV*_*bias*_*/V*_*π*_ represents bias voltage induced phase shift, where *V*_*bias*_ is amplitude of the bias voltage and *V*_*π*_ denotes half-wave voltage of the PolM. Here *φ*_*0*_ is tuned to be 90°. *β = πV*_*RF*_*/V*_*π*_ is modulation index, where *V*_*RF*_ denotes amplitude of the microwave signal, *ω* is angular frequency of microwave signal.

Afterwards, the signal is sent into a PM-FBG through a PC2. The PM-FBG is a key component which can be fabricated by using a uniform grating phase mask. Due to the birefringence effect, it has two separated and orthogonally-polarized transmission profiles along the fast and slow axes in the fiber, i.e. x′ and y′ axes, which is shown at location II of (Fig 1B). By tuning the PC2, the polarization directions of the two wavelengths along x and y axes have an incident angle of 45° relative to the fast and slow axes of the PM-FBG, respectively. The two optical carriers are aligned with the center of the two transmission bands of the PM-FBG. After passing through the PM-FBG, the optical carriers of two signals are suppressed separately in the two orthogonal polarizations. Along the fast axis (x′) of the PM-FBG, the optical carrier at the wavelength *λ*_*2*_ is filtered out but the rest of the modulated signal can be transmitted. Meanwhile, along the slow axis (y′) of the PM-FBG, only the optical carrier at the wavelength *λ*_*1*_ is removed. Thus, the optical field at the output of PM-FBG is

$$\begin{array}{l} E_{\text{II}}=\hat{x'}\left(E_{1}e^{j\omega_{1}t}\sum_{n=-1}^{1}\left[j\sin\theta +{\left(-1\right)}^{n}\cos\theta \right]J_{n}\left(\beta \right)e^{jn\omega t}+E_{2}e^{j\omega_{2}t}\sum_{n=-1,1}\left[j\cos\theta +{\left(-1\right)}^{n}\sin\theta \right]J_{n}\left(\beta \right)e^{jn\omega t}\right)\\ \;+\hat{y'}\left(E_{2}e^{j\omega_{2}t}\sum_{n=-1}^{1}\left[j\cos\theta -{\left(-1\right)}^{n}\sin\theta \right]J_{n}\left(\beta \right)e^{jn\omega t}+E_{1}e^{j\omega_{1}t}\sum_{n=-1,1}\left[j\sin\theta -{\left(-1\right)}^{n}\cos\theta \right]J_{n}\left(\beta \right)e^{jn\omega t}\right) \end{array}$$ (2)

After transmitting through the fiber, the dispersion is introduced to the signal as

$$\begin{array}{l} E=\hat{x'}\left(E_{1}e^{j\omega_{1}t}\sum_{n=-1}^{1}\left[j\sin\theta +{\left(-1\right)}^{n}\cos\theta \right]J_{n}\left(\beta \right)e^{j\left(n\omega t+\Phi_{1,n}\right)}+E_{2}e^{j\omega_{2}t}\sum_{n=-1,1}\left[j\cos\theta +{\left(-1\right)}^{n}\sin\theta \right]J_{n}\left(\beta \right)e^{j\left(n\omega t+\Phi_{2,n}\right)}\right)\\ \;+\hat{y'}\left(E_{2}e^{j\omega_{2}t}\sum_{n=-1}^{1}\left[j\cos\theta -{\left(-1\right)}^{n}\sin\theta \right]J_{n}\left(\beta \right)e^{j\left(n\omega t+\Phi_{2,n}\right)}+E_{1}e^{j\omega_{1}t}\sum_{n=-1,1}\left[j\sin\theta -{\left(-1\right)}^{n}\cos\theta \right]J_{n}(\beta )e^{j\left(n\omega t+\Phi_{1,n}\right)}\right) \end{array}$$ (3)

where *Φ*_*1*, *n*_ *= -n*^*2*^*λ*_*1*_^*2*^*DLω*^*2*^*/4πc* and *Φ*_*2*, *n*_ *= -n*^*2*^*λ*_*2*_^*2*^*DLω*^*2*^*/4πc* are the dispersion-induced phase shifts, *λ*_*1*_ and *λ*_*2*_ are the two wavelengths of the laser, *D* represents chromatic dispersion parameter, *L* denotes the length of fiber, *c* is the speed of light in vacuum.

Finally, the two wavelengths are separated by a WDM and then they are detected by PD1 and PD2. The AC terms of the photocurrents are

$$i_{1}(t)\propto {\left|E_{1}\right|}^{2}J_{0}J_{1}\sin(\Phi_{1,1}-2\theta )\sin\omega t$$ (4)

$$i_{2}(t)\propto {\left|E_{2}\right|}^{2}J_{0}J_{1}\sin(\Phi_{2,1}+2\theta )\sin\omega t$$ (5)

Comparing the microwave powers from the two PDs, the ACF can be derived as

$$ACF=\frac{P_{1}}{P_{2}}=\frac{{\left|E_{1}\right|}^{4}{sin}^{2}(\Phi_{1,1}-2\theta )}{{\left|E_{2}\right|}^{4}{sin}^{2}(\Phi_{2,1}+2\theta )}=\eta \frac{{sin}^{2}(\Phi_{1,1}-2\theta )}{{sin}^{2}(\Phi_{2,1}+2\theta )}$$ (6)

To simplify the calculation, the powers of two wavelength components are set to be the same so that the power ratio *η* = 1.

**Fig 2:**
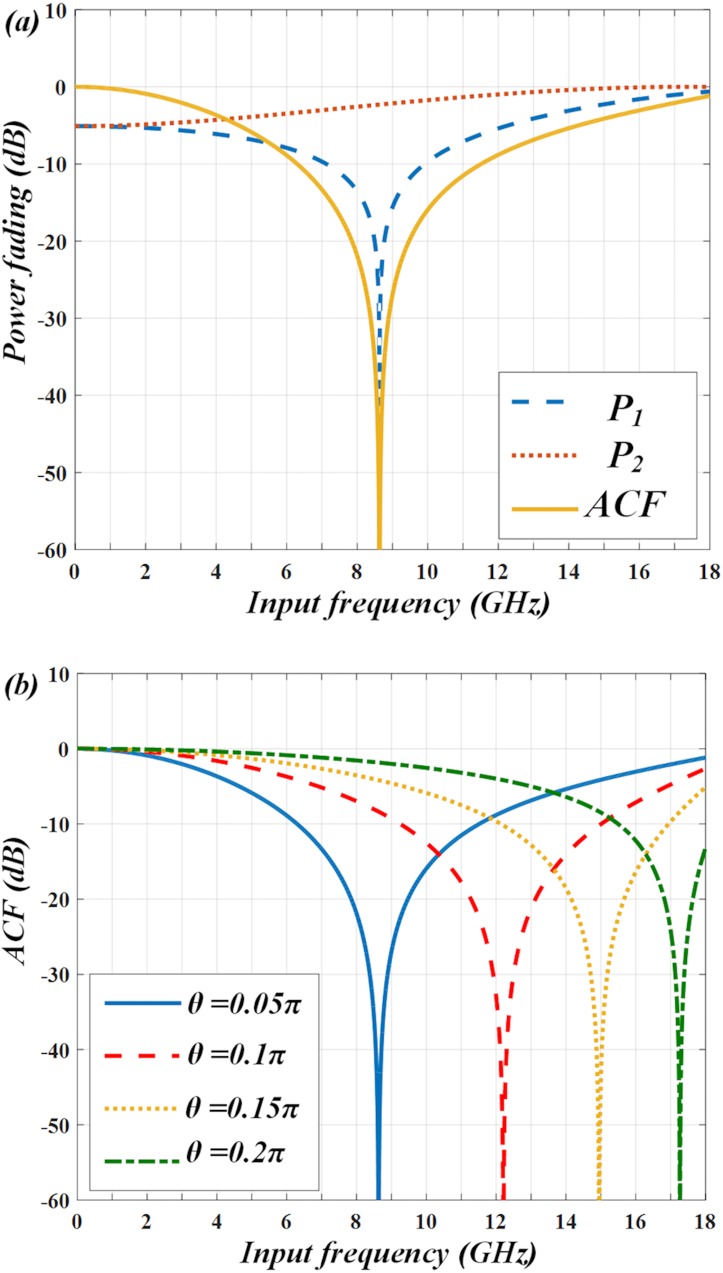
Calculated results. (a) when *θ* = 0.05π, ACF with notch point of 8.5 GHz is derived from two power fading functions P_1_ and P_2_. (b) ACF curve shifts from 8.5 GHz to 12.1 GHz, 14.9 GHz and 17.2 GHz at different polarization angle *θ* = 0.05π, 0.1π, 0.15π and 0.2π.

From Eq (6) we can see, the ACF is dependent on the dispersion-induced phase shifts *Φ*_*1*,*1*_, *Φ*_*2*,*1*_ and polarization angle *θ*. Among these parameters, the dispersion-induced phase shift actually relates to the variables *λ*, *D* and *L*. For a given PM-FBG, the center wavelengths of its two transmission bands are fixed. The two wavelengths (*λ*_1_ and *λ*_2_) of the laser should be also fixed to align the transmission bands of PM-FBG. Taking an example, two wavelengths are set as 1549.8 nm and 1550.2 nm. We use a SMF whose dispersion parameter *D* = 16.75 ps/nm∙km. In order to satisfy the requirements of long-distance transmission, the length of fiber can be as long as *L* = 10 km. Since the polarizations of the two modulated wavelength components are separated by the PM-FBG before transmission in the SMF, the walk off effect in the IFM systems [18] can be eliminated to some extent. Therefore, we can flexibly adjust the ACF curve by tuning *θ*. When *θ* is set to be 0.05π, the power fading of P_1_, P_2_ and the corresponding ACF are plotted in (Fig 2A). As can be seen, the notch point of ACF is corresponding to the frequency of 8.5 GHz. The ACF decreases monotonically from 0 to 8.5 GHz, which infers a measurement range of 0~8.5 GHz. Then by increasing *θ* to be 0.1π, 0.15π and 0.2π, the notch point of ACF shifts to be 12.1 GHz, 14.9 GHz and 17.2 GHz so the measurement range can be extended (as shown in (Fig 2B)).

## Simulation and discussion

**Fig 3:**
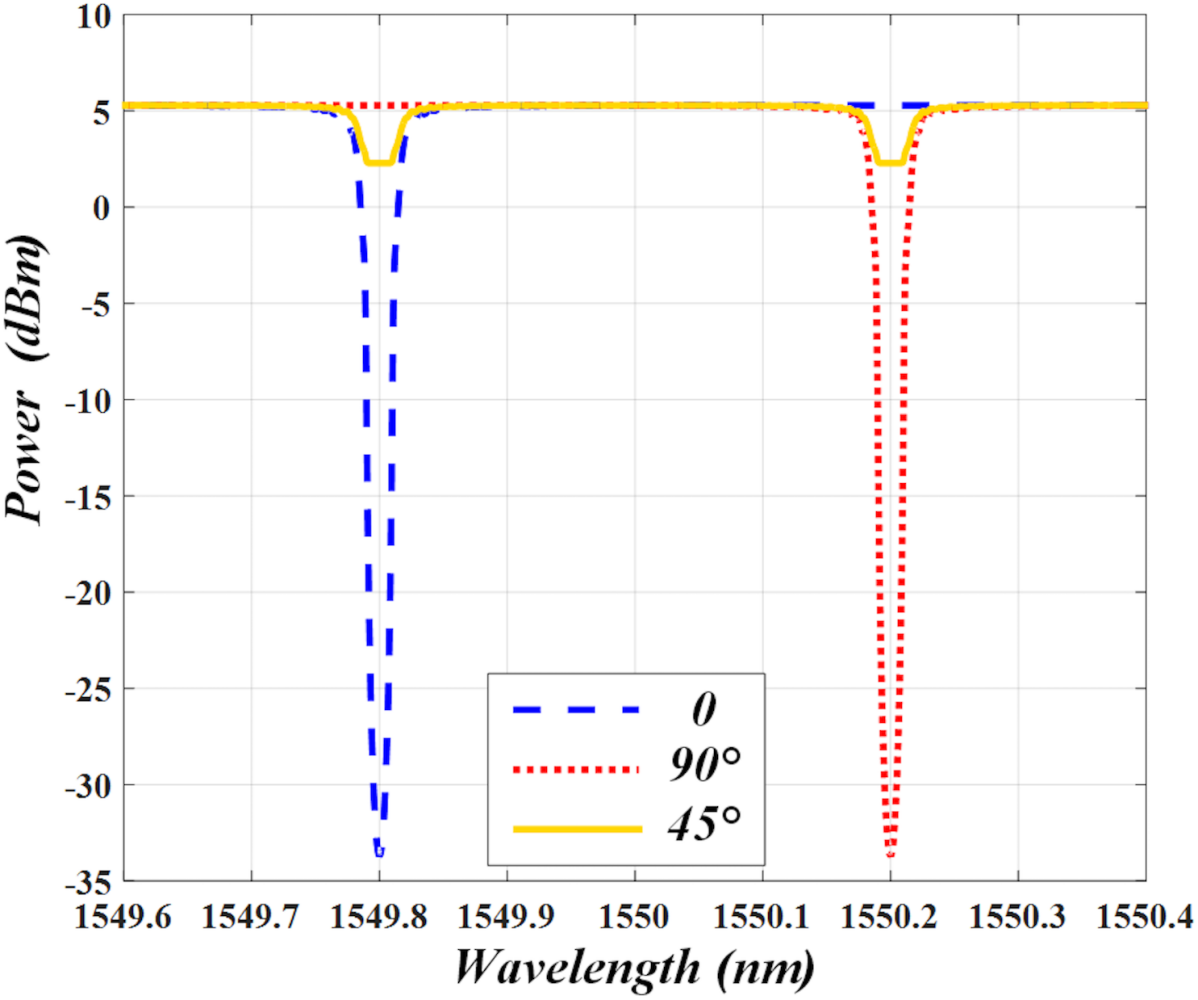
Simulated transmission spectrum of PM-FBG. Transmission spectrum has different profiles when measured at different polarization angles of 0, 90° and 45°.

Simulations are conducted via an OptiSystem 10.0 to verify the proposed IFM system. Firstly, we measure the transmission spectrum of PM-FBG by the use of an un-polarized broadband light source, a linear polarizer (LP) and optical spectrum analyzer (OSA) [22, 23]. The LP is controlled to adjust the incident polarization angle of the input light before PM-FBG. When the angle varies from 0 to 90° and 45° with respect to the fast axis of PM-FBG, the transmission spectrum is shown in Fig 3. As can be seen, it has two orthogonally-polarized transmission bands and the wavelength difference between the two transmission bands is about 0.4 nm.

Then we apply PM-FBG to the IFM system so as to verify its measurement performance. The setup can be found in Fig 1. The laser works at two carrier wavelengths of 1549.8 and 1550.2 nm, so that they are aligned with the center wavelength of PM-FBG separately. The laser linewidth is 0.1 MHz and its two wavelength components are orthogonally-polarized. Then the PC1 is used to adjust the polarization angle and the signal is sent into PolM for complementary phase modulation. The PolM is designed via a Matlab program and a programmable module according to its characteristic. The unknown microwave signal is applied to PolM via its radio frequency port as a driving signal. After that, the principal axes of PM-FBG are aligned with 45° relative to the principal axes of the modulator via the PC2. Next the light waves are transmitted to the 10 km SMF with dispersion parameter *D* = 16.75 ps/nm∙km.

**Fig 4:**
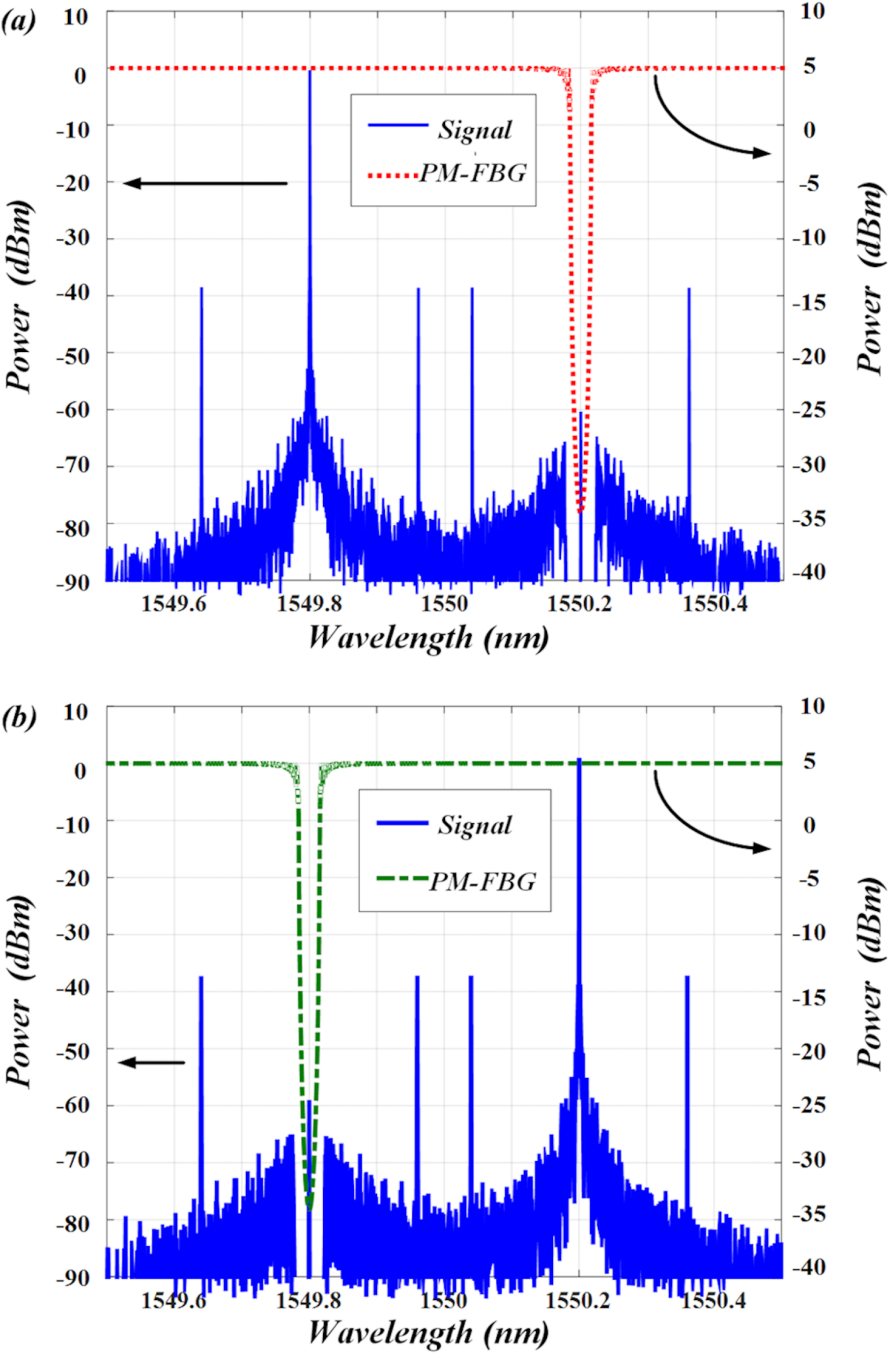
Optical spectrum of the signal and transmission spectrum of the PM-FBG under 45° incident angle. (a) The carrier at *λ*_*2*_ is filtered out by PM-FBG along fast axis. (b) The carrier at *λ*_*1*_ is filtered out by PM-FBG along slow axis.

In order to better illustrate the function of PM-FBG, a polarization beam splitter is connected after the fiber grating, so that the spectra of the output signals in the two orthogonal axes can be observed separately. The transmission profiles of PM-FBG are also shown in Fig 4. As can be seen, along the fast axis, only the optical carrier at *λ*_*2*_ is filtered out (shown in (Fig 4A)). While in (Fig 4B), only the optical carrier at *λ*_*1*_ is filtered out. Therefore, the unwanted frequency components along the two orthogonally-polarized transmission bands of PM-FBG can be removed respectively.

**Fig 5:**
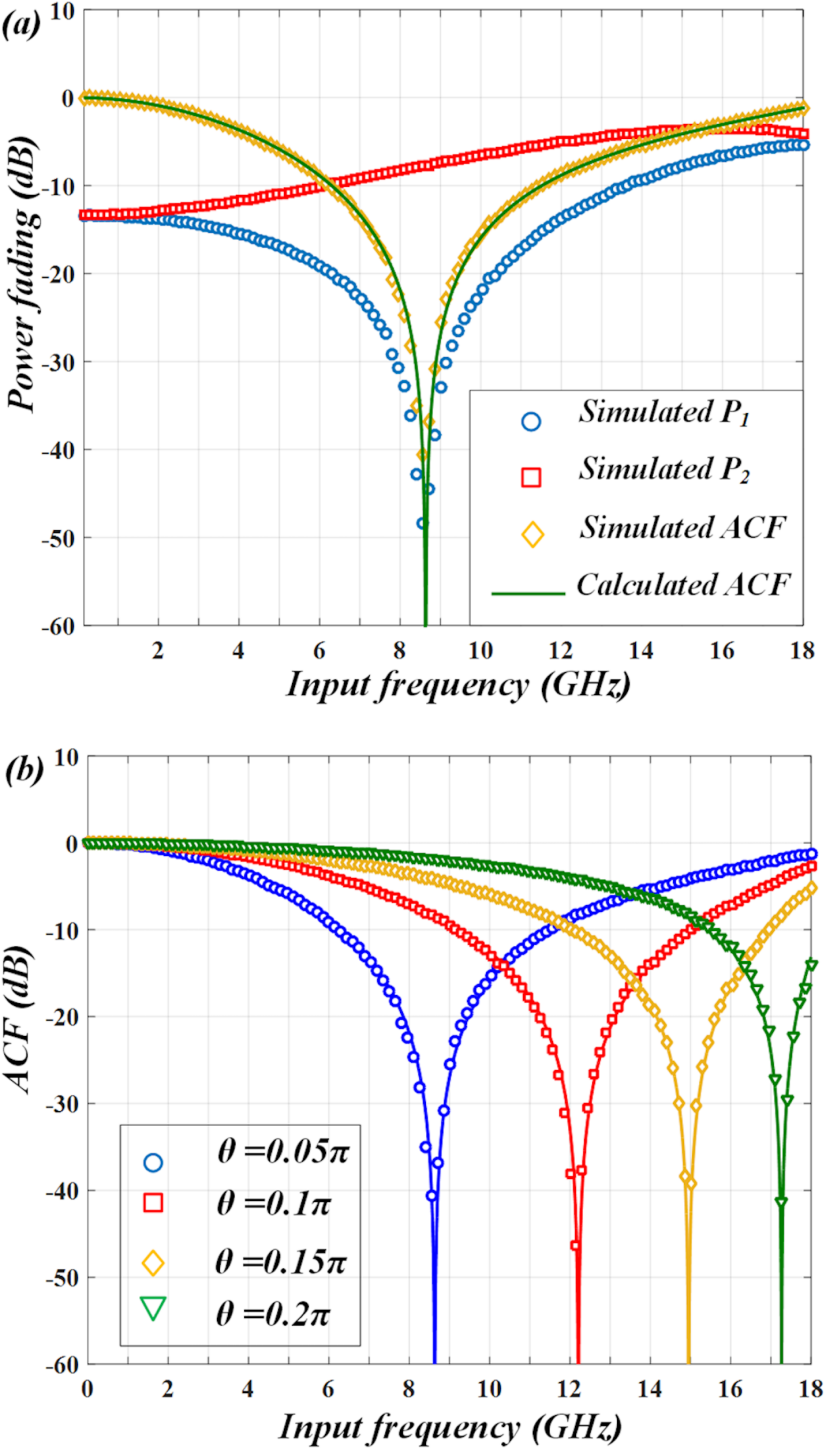
Simulated results. (a) power fading functions and ACF match the calculated results well. (b) Simulated ACFs at *θ* = 0.05π, 0.1π, 0.15π and 0.2π also match the calculations.

By using the two PDs, we can measure the power fading of the two output signals and compare them to obtain the ACF when *θ* = 0.05π. The results are shown in (Fig 5A). It indicates that the simulated (marks) and calculated (lines) results match well. The ACF decreases monotonically between frequencies from 0 to 8.5 GHz. Then polarization angle *θ* is varied from 0.05π to 0.1π, 0.15π and 0.2π and the simulated ACFs are displayed in (Fig 5B). As can be seen, the notch points of the simulated ACFs are shifted from 8.5 GHz to 12.1 GHz, 14.9 GHz and 17.2 GHz as *θ* changes, which also agrees well with the calculation. Thus, the measurement range can be tunable from 0~8.5 GHz to 0~12.1 GHz, 0~14.9 GHz and 0~17.2 GHz in this IFM system.

**Fig 6:**
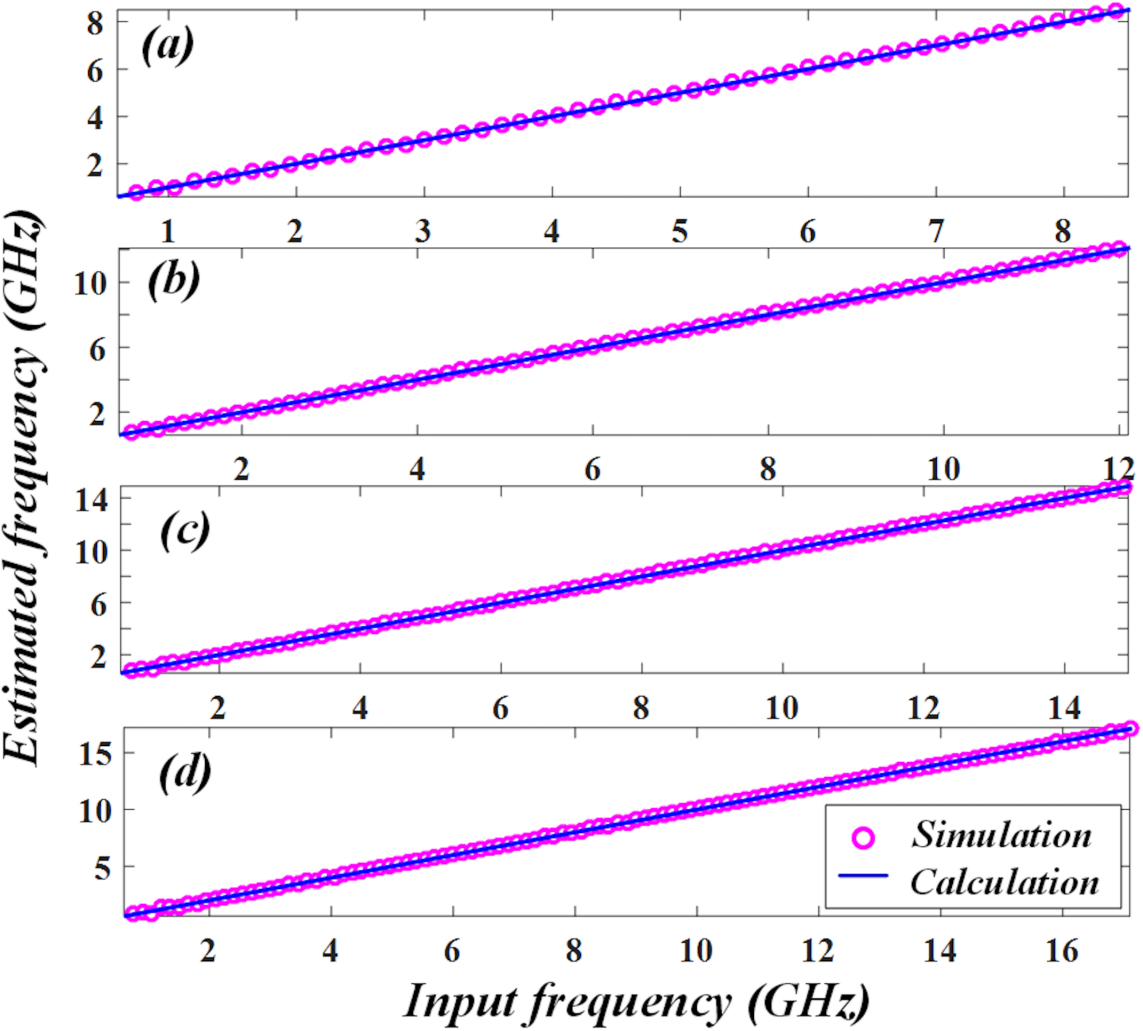
Estimated frequency at different polarization angle *θ*. When *θ* is (a) 0.05π, (b) 0.1π, (c) 0.15π and (d) 0.2π, the estimation roughly fits the calculation and the corresponding measurement range is tuned from 0~8.5 GHz to 0~12.1 GHz, 0~14.9 GHz and 0~17.2 GHz.

Based on the relationship between ACF and input frequency, the unknown frequency can be estimated. The estimation results at different *θ* of (a) 0.05π, (b) 0.1π, (c) 0.15π and (d) 0.2π are shown in Fig 6 respectively. From the figure we can see, the simulated results (dot) roughly fits the calculated results (line) for all the four different measurement ranges, which signifies a high-resolution for the microwave frequency measurement.

**Fig 7:**
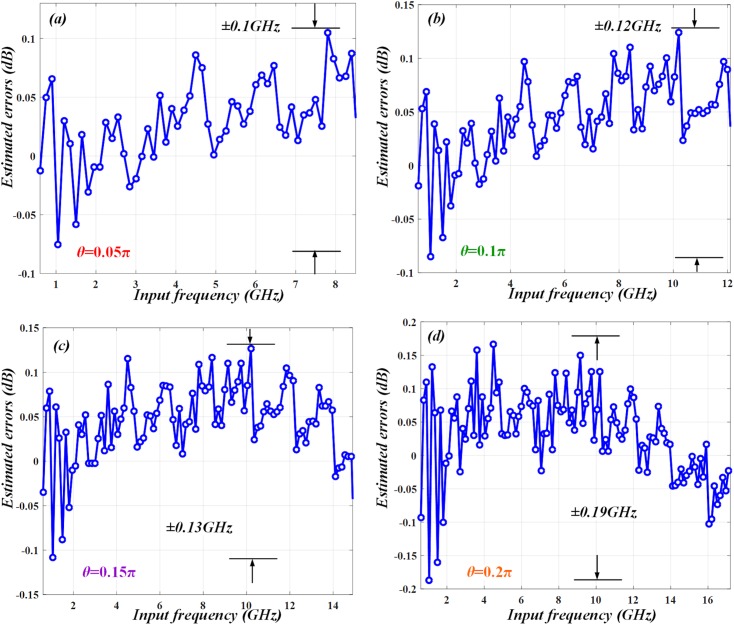
Estimated errors at different polarization angle *θ*. When *θ* changes from (a) 0.05π to (b) 0.1π, (c) 0.15π and (d) 0.2π, the errors increase from ±0.1 GHz to ±0.12 GHz, ±0.13 GHz and ±0.19 GHz.

To further verify the measurement performance by quantitative analysis, we calculate the estimated errors at different polarization angles accordingly. The results are revealed in (Fig 7A)–(Fig 7D). As can be seen, when *θ* = 0.05π, the measurement range is as small as 0~8.5 GHz and the estimated errors are just around ±0.1 GHz. Then by tuning *θ* to 0.1π, 0.15π and 0.2π, the measurement range is stretched to 0~12.1 GHz, 0~14.9 GHz and 0~17.2 GHz. However, the estimated errors gradually increase to ±0.12 GHz, ±0.13 GHz and ±0.19 GHz. It can be found that under a small measurement range, the estimated errors are very small. But with the increment of the measurement range, the slope of ACF at low frequencies becomes flat and the estimated errors become larger. Thus, a trade-off problem between measurement range and resolution exists in the IFM system.

**Fig 8:**
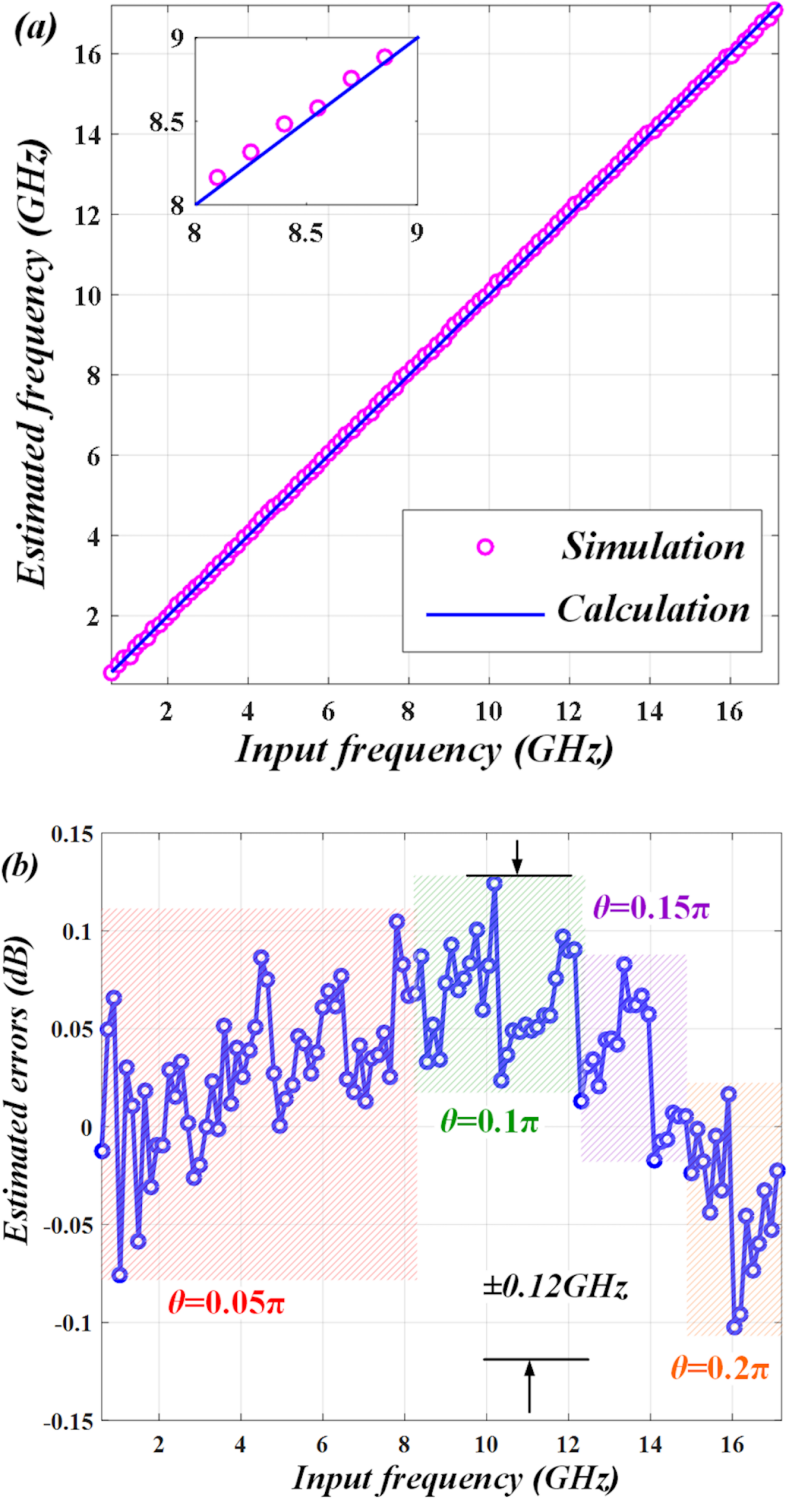
Results of using segmentation measurement. The whole measurement range is divided into four sections (0~8.5 GHz, 8.5~12.1 GHz, 12.1~14.9 GHz, 14.9~17.2 GHz) by tuning *θ* to be 0.05π, 0.1π, 0.15π and 0.2π separately. (a) The estimated frequency matches calculation well. (b) The estimated errors in different sections remain small so that the whole errors maintain within ±0.12 GHz.

In order to address this trade-off problem, we try to employ segmentation measurement to lower the estimated errors. Since the measurement resolution is directly associated with the slope of ACF [4, 16], we can use small *θ* to estimate low frequencies and large *θ* to measure high frequencies. For instance, the whole measurement range can be divided into four different sections (0~8.5 GHz, 8.5~12.1 GHz, 12.1~14.9 GHz, 14.9~17.2 GHz). In each section, the microwave frequencies are estimated at the different *θ* of 0.05π, 0.1π, 0.15π and 0.2π separately. (Fig 8A) illustrates the simulation of estimated frequency, which matches the calculation well. The estimated errors are shown in (Fig 8B). We mark four color regions (red, green, purple and orange) to represent the four sections. In each region, the frequencies are estimated at *θ* = 0.05π, *θ* = 0.1π, *θ* = 0.15π and *θ* = 0.2π separately. It manifests that in the entire measurement range from 0 to 17.2 GHz, the estimated errors can be maintained within around ±0.12 GHz. Thus, the measurement resolution can be effectively improved by using segmentation measurement.

**Fig 9:**
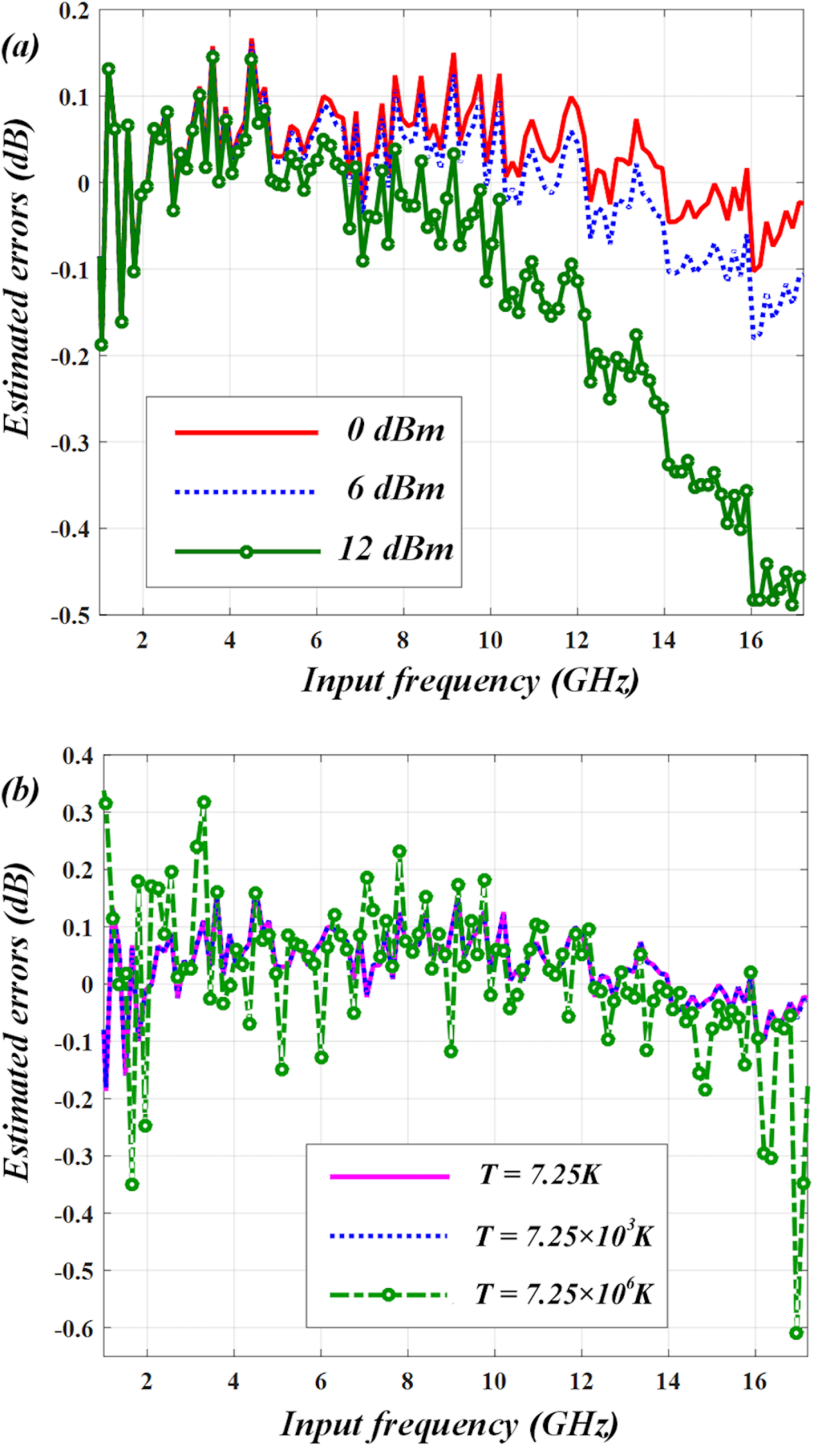
Estimated errors analysis. (a) Varying the laser power has minimal impact on the frequency estimation errors. (b) The photodetector noise can be represented by an equivalent noise temperature, T. The estimated errors are negligible for conventional photodetectors (their equivalent noise temperatures are often below a few thousand degrees Kelvin).

The measurement errors can be attributed to some factors. One is light fluctuation, which may be caused by using an unstable laser source. Therefore, the stability of laser is of significance for the IFM system. To lower the errors, we first use a single-polarization dual-wavelength laser with good stability. Then we verify the impact induced by the variation in average input power via changing the laser power from 0 dBm to 12 dBm. The estimated errors are shown in (Fig 9A). It shows that when the power is smaller than 6 dBm, the measurement errors are within ±0.18 GHz, which can be tolerated. But when the power becomes as large as 12 dBm, the measurement resolution is impaired badly. We also consider the errors caused by thermal noise of PDs and the result is shown in (Fig 9B). Since thermal noise power, P, is directly proportional to absolute temperature, we convert the effect of the photodetector noise to an equivalent noise temperature, T, by using equation P = K_B_TB, where K_B_ is Boltzmann constant and B represents bandwidth. As can be seen, if equivalent noise temperature, T, is as high as 7.25×10^6^ K, the photodetector noise will deteriorate the measurement resolution dramatically, however this is an extremely high equivalent noise temperature; a common photodetector with responsivity of 1 A/W has a noise equivalent power (NEP) of ~20 pW/Hz^1/2^, which corresponds to a noise equivalent temperature of 1.45×10^3^ K. Therefore, the performance of our system is not limited by the photodetector noise in practice. Other effects such as variations of the temperature and bias voltages of the laser and the modulator also influence the accuracy of the system by impacting their stabilities. However, the dual-wavelength laser often works with a good stability at room temperature [19]. The PM fibers can keep a relatively high stability when the temperature variation is controlled within 0.12°C [24]. Furthermore, the voltage variations can be controlled within a small range by using high precision bias control so as to maintain a relatively good performance for the whole IFM system.

## Conclusion

We have proposed a photonic wideband IFM system with tunable measurement range and resolution based on a PM-FBG. The ACF can be adjusted by tuning the polarization angle *θ*, so that the measurement range and resolution are tunable. When *θ* is tuned from 0.05π to 0.1π, 0.15π and 0.2π, the corresponding measurement range can be stretched from 0~8.5 GHz to 0~12.1 GHz, 0~14.9 GHz and 0~17.2 GHz, respectively. In order to alleviate the trade-off problem and improve the measurement resolution, we use segmentation measurement and divide the whole band into four sections. The microwave frequencies are measured in the range of 0~8.5 GHz, 8.5~12.1 GHz, 12.1~14.9 GHz and 14.9~17.2 GHz separately with different *θ*. Thus, a relatively high measurement resolution of ±0.12 GHz can be achieved. It is also found that the impact of some factors such as light fluctuation, photodetector noise, environment temperature and voltage variations can be reduced in our system, so as to ensure a good performance for instantaneous frequency measurement.

This system is affordable and reliable with more consistent characteristics due to the narrowband nature of the optical parts. Using a single-polarization dual-wavelength laser simplifies the architecture of IFM system and enhances the stability against vibration and temperature changes. PM-FBG with high integration also exhibits some promising applications in more polarization manipulating systems.
